# Cancer incidence in British Indians and British whites in Leicester, 2001–2006

**DOI:** 10.1038/sj.bjc.6605744

**Published:** 2010-06-15

**Authors:** R Ali, I Barnes, S W Kan, V Beral

**Affiliations:** 1 Cancer Epidemiology Unit, Nuffield Department of Medicine, University of Oxford, Richard Doll Building, Roosevelt Drive, Oxford, OX3 7LF, UK; 2 INDOX Cancer Research Network, University of Oxford, Old Road Campus Research Building, Oxford, OX, UK

**Keywords:** incidence, ethnicity, migrants, Indians, Hospital Episode Statistics

## Abstract

**Background:**

Incidence rates for many cancers are lower in India than in Britain and it is therefore of interest to compare rates in British Indians to British whites, as well as to rates in India. We present estimates for Leicester, which has the largest population of Indian origin in Britain, and also has virtually complete, self-assigned, ethnicity data.

**Methods:**

We obtained data on all cancer registrations from 2001 to 2006 for Leicester with ethnicity data obtained by linkage to the Hospital Episode Statistics database. Age-standardised incidence rates were calculated for British Indians and British whites as well as incidence rate ratios, adjusted for age and income.

**Results:**

Incidence rate ratios for British Indians compared with British whites were significantly less than 1.0 for all cancers combined (0.65) and for cancer of the breast (0.72), prostate (0.76), colon (0.46), lung (0.30), kidney (0.36), stomach (0.54), bladder (0.48) and oesophagus (0.64), but higher than 1.0 for liver cancer (1.95).

**Conclusion:**

These results are likely to be the most accurate estimate of cancer incidence in British Indians to date and confirm that cancer incidence in British Indians is lower than in British whites in Leicester, particularly for cancer of the breast, prostate, colon and lung (and other smoking-related cancers), but much higher than in India.

There are wide variations in cancer incidence internationally and studies in migrant populations may help explain the relative contribution of genetic and environmental factors to these differences and aid our understanding of cancer aetiology (Parkin, 2004). Incidence rates for many cancers are lower in India than in Britain, with rates for lung, colorectal, breast and prostate cancers (the four most common cancers in the UK) being up to 10 times less common (Ferlay *et al*, 2004). Following large scale migration from India in the 1950s and 1960s (Coleman and Salt, 1996), British Indians now form the largest ethnic minority group in the UK, with more than one million people identifying themselves as British Indian in the 2001 UK census (Office for National Statistics, 2001). It is therefore of interest to compare cancer incidence rates for British Indians to British whites as well as to rates in India.

Such comparisons have been of limited accuracy in the past due to the incomplete ethnicity data held by cancer registries. Since 1995, however, self-assigned ethnicity has been recorded in the Hospital Episode Statistics (HES) database (using the same classification as the census) and HES data can now be linked to cancer registration data thus providing more reliable information on ethnicity than was previously available (Jack *et al*, 2006).

Leicester was chosen for this analysis because it has the largest population of British Indians of any local authority in the UK and has virtually complete ethnicity data recorded in HES. Cancer incidence in South Asians in Leicester has previously been estimated using name analysis methods (Smith *et al*, 2003; Day *et al*, 2009) and here we present estimates using self-assigned ethnicity.

## Materials and methods

The Trent Cancer Registry provided us with data on all cancer registrations from January 2001 to December 2006 for residents of the local authority of Leicester, including individual records with information on sex, age, deprivation and cancer site. The site of the cancer was coded according to the *International Classifications of Diseases*, revision 10.

Ethnicity for each cancer registration was obtained by linkage to the HES database and was available for 97.9% of cancer registrations (6474 out of 6615). Deprivation was assessed using the income domain of the Index of Multiple Deprivation 2007 (IMD 2007) and coded according to national quintiles. The IMD 2007 contains seven domains of deprivation: income, employment, health deprivation and disability, education skills and training, barriers to housing and services, crime and living environment (Department of Communities and Local Government, 2007). We used only the income domain for these analyses as health-related measures might be correlated with cancer incidence.

Population estimates for the local authority of Leicester were obtained from the 2001 census, stratified by sex, age, ethnicity and quintile of the income domain of the IMD 2007. Analyses were performed using STATA and the R statistical software packages.

First, we estimated age-standardised rates of cancer per 100 000 person-years for British Indians and British whites using the 1960 Segi world population, with age at diagnosis of cancer being classified into the following five categories: <45, 45–54, 55–64, 65–74 and >75 years. We then used Poisson regression to estimate incidence rate ratios (comparing British Indians to British whites) adjusting for age and income.

The majority of British Indians in Leicester are of Gujarati origin (about 80%) either having come directly from India or via East Africa (Roberts-Thomson, 2008). We were therefore interested in the cancer incidence rates from registries in Ahmedabad (the capital of Gujarat state) and Mumbai, which has the largest Gujarati population in India outside Gujarat (Office of Registrar General, 2001). The age-standardised rates for the Indian registries are also standardised using the 1960 Segi world population and were obtained from Cancer Incidence in Five Continents (Parkin *et al*, 2002; Curado *et al*, 2007).

The study was approved by the Oxford Research Ethics Committee.

## Results

In 2001 the population of the local authority of Leicester was 279 921 with 72 033 people (26%) identifying themselves as British Indian and 169 455 (61%) as British white (Office for National Statistics, 2001). Demographic information from the 2001 census for British Indians and British whites in Leicester is presented in Table 1. This shows that British Indians are, on average, younger and poorer than the British white population. Just under half (43%) of the British Indians were born in the UK with the majority identifying themselves as Hindu (55%), whereas Muslims (25%) and Sikhs (15%) also form significant minorities.

In total there were 6615 cases of cancer over the period 2001 to 2006 in Leicester. Data on ethnicity were available for 98% of these cases with 4609 (70%) British white and 742 (11%) British Indian, as shown in Table 2. Data on site of cancer, sex, age and income were complete.

### Comparison of British Indians to British whites

The numbers of cases of individual cancers for men and women and estimates of the age-standardised incidence rates for the most common cancers (at least one case per year) in British whites and British Indians are presented in Table 3.

The five most common cancers in British Indians were of the breast (*n*=149), prostate (*n*=63), lung (*n*=53), non-Hodgkin's lymphoma (*n*=41) and rectum (*n*=39) whereas the five most common in British whites were of the lung (*n*=723), breast (*n*=710), colon (*n*=367), prostate (*n*=423) and rectum (*n*=179).

Incidence rate ratios for men and women and both sexes combined, adjusted for age and income, comparing British Indians to British whites are shown in Table 4.

Incidence rate ratios for British Indians compared with British whites were significantly less than 1.0 for all cancers combined and for cancer of the breast, prostate, colon, lung, kidney, stomach, bladder and oesophagus.

Liver cancer was the only cancer in which the incidence rate ratio was greater than 1.0 for British Indians compared with British whites.

### Comparison to Indians in India

Age-standardised incidence rates from the Mumbai and Ahmedabad cancer registries in India are also shown in Table 3. The most common cancers in the Indian registries were of the head and neck, breast, lung, cervix and oesophagus. For the majority of cancers, age-standardised rates for British Indians were higher than for Indians in India, but lower than in British whites.

## Discussion

In this study, we estimated cancer incidence rates for British Indians and whites in Leicester using virtually complete, self-assigned ethnicity data. The age-standardised incidence rate ratios comparing British Indians to British whites showed that British Indians had a significantly lower risk of developing cancer of the breast, prostate, colon, lung, kidney, stomach, bladder and oesophagus as well as all cancers combined. Liver cancer was the only site where British Indians had a significantly higher risk compared with British whites.

Although acknowledging the limitations of the Indian data (see below), our results also suggest that overall cancer incidence in British Indians in Leicester is higher than in both Ahmedabad and Mumbai, mainly due to a higher incidence of breast, prostate, lung and colon cancer. Incidence rates for nearly all cancers in British Indians are closer to the rates in British whites than to Indians in India with breast, prostate, lung and colorectal cancer now also the most common cancers in British Indians. Breast cancer incidence in British Indians is still lower than in British whites but their rates would be expected to increase and approach those in British whites as they adopt the reproductive habits and lifestyle of British whites. Incidence rates in the UK are also increased by screening which of course is not present in India. The higher prostate cancer rates in British Indians compared with Indians in India may also reflect increased surveillance in the UK compared with India as well as the effects of changes in lifestyle. The lower lung cancer rates in British Indians compared with British whites are to be expected given the lower prevalence of smoking in British Indians 20–30 years ago, particularly among women (Balarajan and Yuen, 1986). Similarly, the lower incidence of stomach, bladder, kidney and oesophageal cancer in British Indians is also likely to be due to lower smoking prevalence.

The results for colon cancer are particular interesting as rates in British Indians are still much lower than in British whites, which is in contrast to the experience of other migrant groups (e.g. Japanese migrants to the USA) who were found to have similar rates of colon cancer to white Americans within one generation (McCracken *et al*, 2007). This could be due to dietary factors, as most South Asians in the UK maintain a fairly typical South Asian diet, and there is some evidence that certain constituents of the Indian diet (e.g. spices with high salicylate levels (Paterson *et al*, 2006) and turmeric (Johnson and Mukhtar, 2007) have anti-carcinogenic effects, which, if proven, could have potential use in chemoprevention.

The results for colon cancer are also in contrast to those for rectal cancer for which incidence rates in British Indians were the same as in British whites. Rectal cancer also has a higher incidence than colon cancer in some parts of India, including Ahmedabad (Parkin *et al*, 2002), and there is some evidence that different factors (both genetic and environmental) are important in their pathogenesis (Wei *et al*, 2004; Curtin *et al*, 2009).

The higher incidence of liver cancer in British Indians is likely to be due to the higher prevalence of Hepatitis B and C infection in British South Asians (Hahne *et al*, 2003; Gungabissoon *et al*, 2007) as alcohol intake is generally lower in British Indians than British whites, particularly among women (Erens *et al*, 2000; Sproston, 2006). There is also some evidence that chewing of *paan*, which is much more common in British Asians than British whites, increases the risk of developing liver cancer (Tsai *et al*, 2001). Mortality from hepatocellular carcinoma has also been shown to be higher in those born in India (Bhala *et al*, 2009).

Our study results also suggest that British Indians in Leicester are no longer at increased risk of developing cancer of the head and neck compared with British whites. This finding contrasts with studies from the 1990s in Leicester (Smith *et al*, 2003) and in England (Winter *et al*, 1999) but is in agreement with the most recent national study that covered a similar time period to ours (Forman, 2009). This is likely to be due to the fact that chewing of *paan* by British Indians has decreased over the years, particularly in men (Erens *et al*, 2000; Sproston and Mindell, 2006).

The pattern of cancer incidence we observe in British Indians may also provide an indication of what may happen in the future in certain parts of India (particularly urban areas) as it undergoes its own rapid epidemiological transition. We also note that although the incidence of lung, breast and colon cancer is lower in British Indians than in British whites, rates of diabetes and ischaemic heart disease are higher, even though some of the risk factors are similar (Forouhi *et al*, 2006).

The two previous studies in Leicester (Smith *et al*, 2003; Day *et al*, 2009), as well others in the southeast of England (Jack *et al*, 2009a, 2009b) and in England as a whole (Winter *et al*, 1999; Rastogi *et al*, 2008; Forman, 2009), produced a similar pattern of results to ours, showing lower cancer incidence rates in South Asians compared with non-South Asians for cancer of the lung, colorectal, breast and prostate, and for all cancers combined.

However, we believe that our study is likely to have estimated cancer incidence rates more accurately than these previous studies that were limited by the incomplete ethnicity data held by the cancer registries. The main technique used to try and overcome this problem was to assign ethnicity on the basis of name (Winter *et al*, 1999; Smith *et al*, 2003; Rastogi *et al*, 2008) but this method has significant limitations in comparison to the use of self-assigned ethnicity. For example, we were able to use the same ethnicity measure in the numerator and denominator whereas studies based on name analysis use names to estimate the numerator but self-assigned ethnicity census data for the denominator, leading to possible numerator/denominator mismatch. We were able to look specifically at British Indians whereas name analysis involves grouping all South Asians (Indians, Pakistani and Bangladeshis) together even though there are important differences between them. Name analysis methods also have to group all non-South Asians together (including whites, blacks and Chinese) whereas our method allows us to analyse British whites only. A further limitation of name analysis methods is that the majority of Muslim names are of Arabic derivation and so it is difficult to distinguish South Asian Muslims from Northern African, Arab, Iranian, Turkish and Eastern European Muslims (Nanchahal *et al*, 2001). This is a significant problem in Leicester where about 6000 Muslims are not of South Asian origin and so may have been misclassified. Name analysis methods have also been shown to misclassify a proportion of South Asians as non-South Asians and *vice versa* (Cummins *et al*, 1999; Nanchahal *et al*, 2001). Studies that use country of birth as a proxy for ethnicity (Harding and Rosato, 1999; Wild *et al*, 2006; Harding *et al*, 2009) have also become less useful as the largest proportion of British Indians (and nearly half in Leicester) were born in the UK and some of those born in India are not of Indian ethnicity (Office for National Statistics, 2001).

Previous studies that estimated cancer incidence in British Indians by linkage to HES ethnicity data were limited by the much higher proportions of missing ethnicity data (24–40%) (Forman, 2009; Jack *et al*, 2009a, 2009b). In contrast, our study had virtually complete self-assigned ethnicity data and we were also able to adjust for socioeconomic status which is an important confounder in studies on health and ethnicity (Pollock and Vickers, 1997). We also compared British whites and British Indians in the same geographical location in the UK (i.e. Leicester), which is important as British Indians migrants are not distributed homogenously in the UK with the majority living in a few large urban areas (Office for National Statistics, 2001). There are also known to be geographical variations in cancer incidence in the UK and urban–rural differences (Forman, 2008) and so it is inappropriate to compare disease rates in migrants with the entire population of the host country (Parkin and Khlat, 1996). Our study also compared cancer rates in an Indian migrant population with the area of India from which they originated whereas previous studies having used cancer rates in India as a whole (Winter *et al*, 1999; Rastogi *et al*, 2008). Such national comparisons can be misleading as there is significant variation in cancer incidence rates in different parts of India (Nandakumar *et al*, 2005).

We were unable to analyse Indians by their religion, which would have been useful as there are important differences in lifestyle and culture that could affect the incidence of some cancers (e.g. vegetarianism is much more common in Hindus than Muslims or Sikhs). We also did not have information on whether the British Indians were first or second generation, their age at migration or duration of residence. This would have enabled us to see if rates were higher in those who had been here longer or were born here (and are therefore more likely to be ‘acculturated’ and had greater ‘exposure’ to the UK environment). The comparison of rates between British Indians and India is also limited by the fact that the data from Ahmedabad cover an earlier time period (1993–1997) and there were problems with the quality of the data (Parkin *et al*, 2002). Although the data from Mumbai are more recent (1998–2002), and of better quality, only about 20% of Mumbai's population is of Gujarati origin (Office of Registrar General, 2001). There may also be differences in cancer registration practices and there are certainly differences in access to screening and early detection that will reduce incidence rates particularly for breast and prostate cancer. Rates in India are also generally thought to be underestimated due to under-diagnosis and under-ascertainment, particularly among the elderly and in rural areas (Swaminathan and Sankaranarayanan, 2010). Migrants are also a selective group and may not be representative of the population from which they arose and they may be more or less healthy than the population in their native country (Parkin and Khlat, 1996).

## Conclusion

Our study results are likely to be the most accurate estimate of cancer incidence in British Indians to date and confirm that cancer incidence in British Indians is lower than in British whites, particularly for cancer of the colon, breast, prostate, lung and other smoking-related cancers.

We have also shown that cancer incidence in British Indians is much higher than for Indians in India (and that rates for nearly all cancers in British Indians are closer to the rates in British whites than to Indians in India) consistent with the hypothesis that it is changes in environmental factors that are mainly responsible for the changes in cancer incidence, although there may also be differences in genetic susceptibility.

## Figures and Tables

**Table 1:** Comparison of demographic characteristics of British whites and British Indians in Leicester (Office for National Statistics, 2001)

	**British Indians**	**British whites**
**Characteristic**	**Number (%)**	**Number (%)**
*Sex*		
Male	34 977 (49)	80 892 (48)
		
*Age*		
<44	52 190 (73)	10 5873 (63)
45–54	9671 (13)	18 863 (11)
55–64	5220 (7)	15 091 (9)
65–74	3206 (5)	14 025 (8)
75+	1747 (2)	15 604 (9)
		
*Income quintile*		
1 (most deprived)	41 527 (58)	80 517 (48)
2	18 017 (25)	38 492 (23)
3	9025 (13)	30 428 (18)
4	2733 (4)	15 415 (9)
5 (least deprived)	732 (1)	4604 (3)
		
*Country of birth*		
UK	31 271 (43)	16 6549 (98)
India	23 220 (32)	168 (0.1)
Other	17 548 (24)	2738 (2)
		
*Religion*		
Christian	762 (1)	10 9249 (65)
Hindu	39 517 (55)	198 (0.1)
Muslim	18 180 (25)	471 (0.3)
Sikh	10 536 (15)	147 (0.1)
Other/Not stated	3038 (4)	14 346 (35)

**Table 2:** Distribution of the population, and of registered cancers in Leicester by ethnic group

**Ethnic group**	**Population (%)**	**Number of registered cases of cancer (%)**
British white	16 9456 (60.6)	4609 (69.7)
British Indian	72 033 (25.7)	742 (11.2)
All other ethnic groups (black, Chinese, other Asian, other white and mixed)	38 432 (13.7)	1123 (17.0)
No ethnicity recorded	0	141 (2.1)
Total	27 9921 (100)	6615 (100)

**Table 3:** Age-standardised cancer incidence rates for (a) male and (b) female British whites and British Indians in Leicester, and Mumbai and Ahmedabad, India

		**British whites in Leicester (2001–2006)**		**British Indians in Leicester (2001–2006)**		**Age-standardised rates**	
**ICD code**	**Site**	**No.**	**Age-standardised rates (95% CI)**	**No.**	**Age-standardised rates (95% CI)**	**Mumbai (1998–2002)**	**Ahmedabad (1993–1997)**
(a)							
C00–C14/C30–32	Head and neck	103	15.0 (11.9–18.1)	28	13.9 (8.7–19.1)	26.2	29.5
C15	Oesophagus	94	11.4 (8.9–13.9)	11	5.1 (2.1–8.2)	6.7	8.0
C16	Stomach	114	12.0 (9.6–14.5)	12	6.0 (2.6–9.5)	4.6	2.1
C18	Colon	174	18.2 (15.2– 21.3)	20	9.9 (5.5–14.2)	3.0	1.9
C19–20	Rectum	101	11.7 (9.2–14.2)	20	9.8 (5.5–14.1)	2.6	2.3
C22	Liver	21	2.6 (1.4–3.9)	9	4.6 (1.6–7.6)	4.5	1.9
C25	Pancreas	56	6.2 (4.4–8.0)	10	4.6 (1.7–7.6)	2.2	1.1
C33–34	Lung	440	50.4 (45.2–55.5)	41	21.0 (14.5–27.4)	9.7	11.4
C61	Prostate	423	45.8 (41.0–50.6)	63	32.8 (24.7–41.0)	6.9	3.6
C64–66/C68	Kidney	86	11.7 (9.0–14.3)	10	5.2 (2.0–8.5)	2.0	1.2
C67	Bladder	108	11.4 (9.0–13.8)	11	5.5 (2.2–8.8)	3.8	2.5
C70–72	Brain	45	7.5 (5.2–9.9)	8	3.8 (1.2–6.6)	3.7	2.5
C81	Hodgkin's disease	11	2.3 (0.9–3.7)	9	4.1 (1.4–6.9)	0.9	0.7
C82–85/C96	Non-Hodgkin's lymphoma	76	11.1 (8.4–13.8)	20	10.2 (5.7–14.7)	4.4	2.5
C91–95	Leukaemia	53	6.7 (4.7–8.7)	7	3.4 (0.9–6.0)	4.4	3.1
	All sites (excluding non-melanoma skin cancer)	2219	266.3 (254.0–278.7)	330	165.4 (147.4–183.4)	100.9	105.8
(b)							
C00–C14/C30–32	Head and neck	41	4.5 (2.9–6.2)	17	7.0 (3.7–10.4)	9.4	7.7
C15	Oesophagus	47	3.9 (2.5–5.2)	9	3.9 (1.3–6.5)	4.6	4.1
C16	Stomach	54	4.6 (3.1–6.1)	8	3.2 (1.0–5.5)	2.3	1.3
C18	Colon	193	15.5 (12.8–18.2)	14	5.9 (2.8–9.0)	2.4	1.3
C19–20	Rectum	78	6.5 (4.7–8.3)	19	7.8 (4.3–11.4)	1.8	1.5
C22	Liver	18	1.5 (0.6–2.3)	8	3.4 (1.0–5.8)	2.2	0.5
C25	Pancreas	65	5.5 (3.9–7.2)	6	2.3 (0.4–4.1)	1.6	0.8
C33–34	Lung	313	29.8 (25.9–33.8)	12	4.9 (2.1–7.6)	3.1	2.5
C50	Breast	710	88.6 (81.1–96.1)	149	63.0 (52.8–73.2)	26.9	19.1
C53	Cervical	65	12.2 (9.1–15.3)	17	7.6 (4.0–11.3)	14.5	13.4
C54–55	Uterus	135	16.6 (13.4–19.8)	30	13.1 (8.4–17.8)	3.8	1.8
C56	Ovarian	95	13.0 (10.1–16.0)	29	12.6 (8.0–17.2)	7.1	3.6
C70–72	Brain	27	3.2 (1.8–4.6)	7	3.2 (0.8–5.6)	2.8	1.5
C73	Thyroid	19	3.0 (1.5–4.5)	7	3.0 (0.8–5.2)	1.5	0.8
C82–85/C96	Non-Hodgkin's lymphoma	89	8.9 (6.7–11.0)	21	9.1 (5.2–13.0)	2.9	1.2
C91–95	Leukaemia	31	3.0 (1.7–4.4)	10	4.4 (1.6–7.4)	3.2	2.5
	All sites (excluding non-melanoma skin cancer)	2390	258.9 (246.5–271.4)	412	175.1 (158.0–192.2)	103.7	81.8

Abbreviation: ICD=*International Classifications of Diseases*.

All rates are standardised to the age distribution of the Segi standard population.

**Table 4:** Incident rate ratios in British Indians compared with British whites in Leicester

		**Incidence rate ratio (95% CI) adjusted for age and income**			***P*-value for**
**ICD code**	**Cancer site**	**Males**	**Females**	**Both sexes**	**both sexes** a
C00–C14/ C30–32	Head and Neck	0.89 (0.58–1.36)	1.72 (0.95–3.13)	1.09 (0.77–1.55)	0.6
C15	Oesophagus	0.49 (0.26–0.92)	0.97 (0.47–2.03)	0.64 (0.40–1.03)	0.05
C16	Stomach	0.46 (0.25–0.84)	0.69 (0.31–1.47)	0.54 (0.34–0.87)	0.006
C18	Colon	0.52 (0.33–0.84)	0.38 (0.22–0.67)	0.46 (0.32–0.66)	<0.001
C19–20	Rectum	0.83 (0.51–1.35)	1.19 (0.71–2.01)	0.98 (0.69–1.40)	0.9
C22	Liver	1.68 (0.74–3.77)	2.34 (0.98–5.59)	1.95 (1.08–3.54)	0.03
C25	Pancreas	0.82 (0.41–1.64)	0.52 (0.22–1.23)	0.68 (0.40–1.15)	0.1
C33–34	Lung	0.40 (0.29–0.55)	0.16 (0.09–0.29)	0.30 (0.23–0.40)	<0.001
C50	Breast	—	0.72 (0.60–0.86)	—	<0.001
C53	Cervical	—	0.62 (0.36–1.07)	—	0.07
C54–55	Uterus	—	0.77 (0.51–1.16)	—	0.2
C56	Ovarian	—	0.95 (0.62–1.46)	—	0.8
C61	Prostate	0.76 (0.58–0.99)	—	—	0.04
C64–66/C68	Kidney	0.42 (0.22–0.83)	0.15 (0.20–1.10)	0.36 (0.19–0.68)	<0.001
C67	Bladder	0.50 (0.27–0.94)	0.39 (0.12–1.27)	0.48 (0.27–0.84)	0.005
C70–72	Brain	0.50 (0.23–1.08)	1.00 (0.42–2.36)	0.66 (0.38–1.17)	0.1
C73	Thyroid		1.07 (0.43–2.61)	1.16 (0.52–2.59)	0.7
C81	Hodgkin's disease	1.85 (0.75–4.53)	1.87 (0.58–5.99)	1.84 (0.90–3.74)	0.1
C82–85/C96	Non-Hodgkin's lymphoma	0.83 (0.50–1.38)	1.04 (0.63–1.70)	0.93 (0.65–1.32)	0.7
C88–90	Myeloma	0.75 (0.29–1.98)	0.94 (0.36–2.49)	0.84 (0.43–1.68)	0.6
C91–95	Leukaemia	0.50 (0.22–1.11)	1.40 (0.66–2.97)	0.81 (0.47–1.38)	0.4
	All sites (excluding non-melanoma skin cancer)	0.61 (0.54–0.69)	0.67 (0.61–0.76)	0.65 (0.60–0.71)	<0.001

Abbreviation: ICD=*International Classifications of Diseases*.

a *P*-values are for males and females combined except for breast, prostate and gynaecological cancers.
